# The tissue microarray data exchange specification: A document type definition to validate and enhance XML data

**DOI:** 10.1186/1472-6947-5-12

**Published:** 2005-05-04

**Authors:** David G Nohle, Leona W Ayers

**Affiliations:** 1 The Mid-Region AIDS and Cancer Specimen Resource (ACSR), Department of Pathology, The Ohio State University, Columbus, OH USA

## Abstract

**Background:**

The Association for Pathology Informatics (API) Extensible Mark-up Language (XML) TMA Data Exchange Specification (TMA DES) proposed in April 2003 provides a community-based, open source tool for sharing tissue microarray (TMA) data in a common format. Each tissue core within an array has separate data including digital images; therefore an organized, common approach to produce, navigate and publish such data facilitates viewing, sharing and merging TMA data from different laboratories. The AIDS and Cancer Specimen Resource (ACSR) is a HIV/AIDS tissue bank consortium sponsored by the National Cancer Institute (NCI) Division of Cancer Treatment and Diagnosis (DCTD). The ACSR offers HIV-related malignancies and uninfected control tissues in microarrays (TMA) accompanied by de-identified clinical data to approved researchers. Exporting our TMA data into the proposed API specified format offers an opportunity to evaluate the API specification in an applied setting and to explore its usefulness.

**Results:**

A document type definition (DTD) that governs the allowed common data elements (CDE) in TMA DES export XML files was written, tested and evolved and is in routine use by the ACSR. This DTD defines TMA DES CDEs which are implemented in an external file that can be supplemented by internal DTD extensions for locally defined TMA data elements (LDE).

**Conclusion:**

ACSR implementation of the TMA DES demonstrated the utility of the specification and allowed application of a DTD to validate the language of the API specified XML elements and to identify possible enhancements within our TMA data management application. Improvements to the specification have additionally been suggested by our experience in importing other institution's exported TMA data. Enhancements to TMA DES to remove ambiguous situations and clarify the data should be considered. Better specified identifiers and hierarchical relationships will make automatic use of the data possible. Our tool can be used to reorder data and add identifiers; upgrading data for changes in the specification can be automatically accomplished. Using a DTD (optionally reflecting our proposed enhancements) can provide stronger validation of exported TMA data.

## Background

### ACSR

The National Cancer Institute's (NCI) Division of Cancer Treatment and Diagnosis (DCTD) founded the AIDS Malignancy Bank (AMB) in 1994. Now funded as the AIDS and Cancer Specimen Resource (ACSR), a wide variety of biological samples from HIV/AIDS related malignancies are offered to approved researchers [1]. Tissue microarrays (TMA) with hundreds of HIV infected tissue cores [2] are constructed and offered for distribution by the ACSR. To facilitate researchers' review of available TMAs, digital images of tissue cores and accompanying de-identified data are displayed on the ACSR Mid-Region web site .

### API TMA DES

The April 2003 Association for Pathology Informatics (API) TMA Data Exchange Specification (TMA DES) proposes a community-based, open source tool in a common XML format for improved portability of TMA data. A validator (written in Perl) is also provided with the specification (the workshop participants did not approve a DTD and emphasized that users must be allowed to add their own tags) [3]. The usefulness of TMAs to the scientific community is greatly increased by an organized, common approach to producing, navigating and publishing the large quantity of generated data. Exporting TMA data into this API specification format offers an opportunity to evaluate the specification and the validator. Importing and using TMA data from a variety of other institutions also offers an opportunity to further evaluate the utility and functionality of the proposed TMA DES.

### XML

"XML, the Extensible Mark-up Language, is a W3C-endorsed standard for document mark-up. XML is a metamark-up language for text documents. Data is included in XML documents as strings of text. The data is surrounded by text mark-up that describes the data [4]." The tag delimiters that will be used as mark-up are defined in a particular application and comprise a particular language.

The API specification defines a mark-up language for the TMA data representation application. Our identifier for a tissue microarray block is TA00-050, a data text string. The API TMA DES tag delimiter for such a block identifier is block_identifier [6]. The marked up data is an XML element:

<block_identifier>

TA00-050

</block_identifier>

XML data may be well formed or valid. Well-formed data has matching end tags that are properly nested and follows generic XML rules. It may not necessarily be valid, i.e. follow the rules of a particular language. To assure that data is valid, it should be validated more rigorously using a DTD. Although a Perl script validator was published with the TMA DES, XML validation must involve a DTD [5].

### DTD

A document type definition (DTD) is used to specify a valid language of XML elements. The DTD can be used with various validators [6,7] to check that XML documents written in this language are valid. Although a Schema is an alternative way to specify the properties of an XML document, the DTD syntax was part of early XML standards and has long been supported by various XML processors.

In the DTD language, elements are the tag names that will appear between '<' and '>' in XML documents. Entities are temporary names that will not be used as tags but stand for the text in their definition. They may be referred to preceding the name with '&' and following it with ';'. Elements and/or entities are separated by ',' which means followed by or '|' which means or. Parentheses are used to group elements and entities. A suffix can follow groups, elements or entities: '?' means zero or one, '+' means one or many, and '*' means zero or many. Absence of a suffix indicates that a single one is mandated.

This document type declaration (not to be called a DTD although it may contain definitions between the square brackets) defines a block element that must contain a single block_identifier element and may be followed by a single block_description element and any number of core elements:

<!DOCTYPE block [

<!ELEMENT block_identifier (#PCDATA)>

<!ELEMENT block_description (#PCDATA)>

<!ELEMENT core (#PCDATA)>

<!ELEMENT block (block_identifier,

block_description?, core*)>

]>

The #PCDATA (parsed character data) type allows raw text (but not tags or child elements) to be included in an element. The definition portion could appear at the beginning of an XML file (an internal DTD). An external DTD file, block.dtd, containing such a definition could be referred to with the following declaration in the beginning of an XML file:

<!DOCTYPE block SYSTEM "block.dtd">

**Figure 1:**
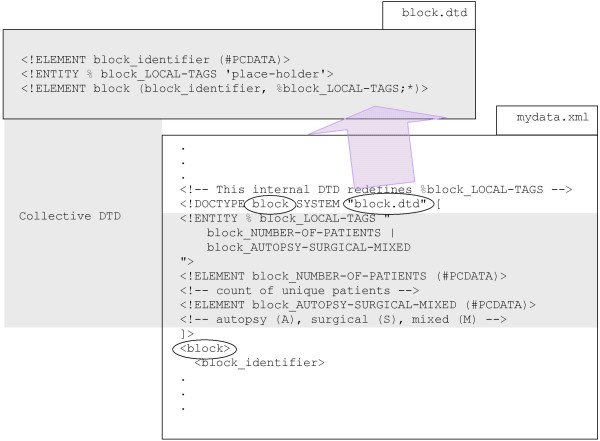
**Document type definition subsets **A DTD can have either an external portion (or subset) or an internal portion or both. Together, these subsets are regarded as the complete DTD. The external DTD subset is in an XML file (or group of files) by itself. The internal DTD subset is placed in the beginning of the XML file within the document type declaration before the root element start tag. The document type declaration refers to the file containing the external DTD subset file name (block.dtd in this case) and contains the DTD name (block in this case) which must be the top level element in the XML file.

When an external DTD file is referred to and an internal DTD subset is present in an XML file, the two parts collectively are the DTD as shown in Figure 1.

The DTD syntax allows definitions to be placed in a file and included into the internal portion as well. In the following example, block.dtd contains the external portion of the DTD and myBlockTags.dtd contains most of the internal portion:

<!DOCTYPE block SYSTEM "block.dtd" [

<!ELEMENT % myBlockTagFile SYSTEM "myBlockTags.dtd">

%myBlockTagFile;

]>

The first definition of a given element or entity encountered is used. Internal DTD definitions are encountered before external ones.

## Implementation

The ACSR has been improving the organization of generated TMA data for several years [8-10]. A sample TMA export illustrates the TMA DES format [See TA00-050.xml in Additional file 1]. Compare this to an improved version for the same TMA during the discussion below [See TA00-050_recordered.xml in Additional file 1].

### Designing the DTD

The XML document type definition (DTD) for the TMA DES must represent the rules given in the specification and the 80 CDEs defined in the associated Tissue MicroArray Common Data Elements document. This DTD [See tmades.dtd in Additional file 1] accommodates the following special circumstances:

• The TMA DES permits locally needed data element (LDE) definitions to extend those defined in it. A thorough DTD must specify every element. When such elements are added, definitions must be added to the DTD locally. The next section, Extending the DTD, explains this further.

• The order of elements within a parent element is never constrained while the presence of at least one of certain CDEs is mandated. The DTD language will not necessarily allow specification of a certain number of elements when the order of elements is unconstrained. The DTD must be deterministic, i.e. a parser looking at successive elements must have only one path forward through the rules. The section called Mandating CDEs discusses this further.

• The TMA DES specifies a hierarchical nesting arrangement for the CDEs defined in it, it does not define the specific values for them. The content of elements is therefore specified as parsed character data (#PCDATA).

### Extending the DTD

Our DTD provides for extensions to the specification to add data elements not in the specification. We made DTD extensions to implement those needed at the Mid-Region ACSR, which serve as examples of how to implement the TMA DES.

Our design uses a single external DTD file with selectable modes to contain definitions that implement the API proposed TMA DES and our proposed improvements and additions to it. We defined three place holder entities (block_LOCAL-TAGS, core_LOCAL-TAGS, slide_LOCAL-TAGS) that can be redefined in the internal portion to extend the specification with local defined elements (LDE) [See myLDEs.dtd in Additional file 1]. We illustrate how to place these in a separate file and include it into multiple XML files to reduce maintenance.

Suppose that age and gender are tracked for cores in TMA blocks at an institution. There are no CDEs in the specification to hold this data. These internal LDE and entity definitions can be shared by several TMA blocks because they are placed in a file called myCoreTags.dtd:

<!ELEMENT core_AGE (#PCDATA)>

<!ELEMENT core_GENDER (#PCDATA)>

<!ENTITY % core_LOCAL-TAGS (core_AGE | core_GENDER)>

This external DTD file fragment defines core_LOCAL-TAGS as a place-holder:

<!ELEMENT core_array-id (#PCDATA)>

<!ELEMENT core (#PCDATA)>

<!ENTITY % core_LOCAL-TAGS (place-holder)>

<!ELEMENT core (core_array-id, (%core_STD-TAGS; |

%core_LOCAL-TAGS;)*>

Multiple XML files, as in the following example, can reference the public external DTD file as well as the locally shared internal DTD file eliminating maintenance of multiple copies. (Usually a public URI is specified: . The file name is used here to simplify the example.)

<!DOCTYPE histo SYSTEM "tmades.dtd" [

<!ENTITY % noLDEs 'IGNORE' >

<!ENTITY % myCoreTagFile SYSTEM "myCoreTags.dtd">

%myCoreTagFile;

]>

**Figure 2:**
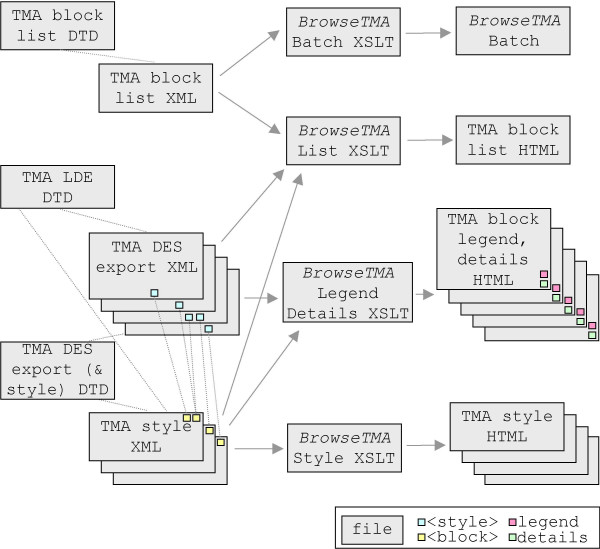
**Implementation **A DTD governs the allowed data elements in each type (TMA block list, TMA LDE, TMA DES export & style) of XML file. The TMA block list uses *BrowseTMA *to process each listed block file and style. *BrowseTMA *produces a single HTML TMA block list file, a TMA block HTML file for each specified block and an HTML TMA style file for each style used.

The first (internal) definition encountered is used. The external definition of block_LOCAL-TAGS as a place-holder is thus ignored when an internal one is provided. A similar arrangement is shown in Figure 2.

TMA DES export (& style) DTD is always tmades.dtd, TMA LDE DTD could be myCoreTags.dtd and TMA DES export XML would then contain the above document type declaration.

### Mandating CDEs

In the following examples, assume there is an entity called header_unlimited_items that contains all of the elements in the header except filename as follows:

<!ENTITY % header_unlimited_items "(

Title |

Creator |

Subject |

Keywords |

Description |

Publisher |

Contributer |

Date |

Resource_Type |

Format |

Resource_Identifier |

Source |

Language |

Relation |

Coverage |

Rights_Management)">

Here is an example of a problem situation. There is a list of CDEs that may appear inside the header CDE. The filename CDE is in this list and has a maximum occurrence of one. All other CDEs in this list have a maximum occurrence that is unlimited. The order of elements is not limited and no CDEs are required. Here is a straightforward way to describe this in a DTD:

<!ELEMENT header (

(%header_unlimited_items;)*,

filename?,

(%header_unlimited_items;)*)>

Stated in English: a header is zero or many of the non-filename elements followed by a single optional filename followed by perhaps more non-filename elements. Although this faithfully describes our situation, a parser will decide this definition is non deterministic because for some input there are multiple paths through the definition (ex. when there are two Title elements and no others, the term before and/or after the filename can be used for them).

The following definition faithfully represents the allowable header contents and is deterministic but is not as easy to understand:

<!ELEMENT header (

(filename, ((%header_unlimited_items;)*)) |

(((%header_unlimited_items;)+),

((filename, ((%header_unlimited_items;)*)))?))>

Stated in English: a header is either:

• a filename followed by zero or many of the non-filename elements or

• this sequence:

◦ one or many of the non-filename elements followed by

◦ zero or one of this sequence:

▪ a filename followed by

▪ perhaps more non-filename elements.

If the language is changed slightly to say that any number of all header elements is allowed a simple definition is possible:

<!ELEMENT header (((%header_unlimited_items;)*))>

Likewise, if a variety of numbers of header elements is necessary (with some restricted differently than others) but it is acceptable to mandate an order, a simple definition is possible:

<!ELEMENT header (filename, Title*, Creator+, Subject?, ...)>

There are other elements that have a situation similar to the header:

• A tma is required to have one or more headers and one or more blocks but the order is unconstrained.

• A block is required to have one or more slide elements, one or more core elements and optionally any number of (non-parent) block elements in an unconstrained order. (In fact, the requirement for at least one slide and one core may be only for the entire file and not per block. A very convoluted DTD would be required to support a per file restriction.)

A DTD that facilitates validation can also have a role in communicating the details of what is acceptable in the XML application language. While allowing as much flexibility as possible so as to not restrict the style that TMA users might like to employ is desirable, simplifications that make the language easier to understand are also desirable and at times may be involved in a trade-off.

### Improving the DTD

While the DTD implements the specification as initially proposed, some improvements were added for conditional use based on the suggestions in [10]. The external DTD can be used in one of two modes by defining one to INCLUDE and the other to IGNORE:

unimproved (default) mode – Enforces the TMA DES rules as proposed, notably:

• Allows multiple header elements within a tma which can be interspersed with block elements within that tma. Ambiguous situations are possible: As each header can have a filename element, what would it mean to have multiple filenames for a single file? Should we associate a certain header with a certain block? How could we tell which?

• Allows at most one filename element to be anywhere within header, if present. The DTD is more difficult to encode and understand unless the filename must be first (or must be an attribute).

• None of these identifiers are required: filename in header, block_identifier in block, slide_identifier in slide, or core_array-id in core. This leaves no certain way to refer to files, blocks, slides and cores. The absence of core_array-id leaves no certain way to locate the core in the array.

• Enforces that at least one block, one slide, and one core element are required. These elements may be empty and serve no purpose. For example, when a TMA block is constructed and no slides have been made from it and it is being provided with exported data to another institution, a slide element must be in the export although no slide exists. Enforcing the presence of these elements does not assure better use of the specification and that data is indeed provided.

improved mode – Enforces the TMA DES rules with the following changes:

• Enforces that there can be at most a single header element within a tma which must precede all block elements within that tma.

• Enforces that filename is the first element within header, if present.

• Enforces that a single identifier is present as the first element within each parent (block_identifier in block, slide_identifier in slide, core_array-id in core). Human readability is improved if an identifier is at the beginning of each element.

• No block, slide, or core elements are required. It is expected that every block, slide and core for which data is to be provided will have a corresponding element containing that data. This expectation does improve usage of the specification.

A program, *BrowseTMA *Reorder XSLT script [see BrowseTMAReorder.xsl in Additional file 1], was added to *BrowseTMA *that can convert unimproved TMA DES XML data to improved data by reordering elements and, if needed, adding consecutively numbered identifiers. Several batch and java scripts are used to invoke the XSLT script [see BrowseTMAReorder.bat in Additional file 1], repair the XML file [see fixordered.js in Additional file 1] and invoke the Microsoft XSLT Parser [see xsltTest.js in Additional file 1].

The same DTD also contains the TMA style definitions. By default they are ignored; defining addStyles as INCLUDE will cause them to be available.

## Results

### Verifying XML data

We used our DTD with the Internet Explorer MSXML2 4.0 parser (set to verify) and at two public web sites to verify a few TMA DES block files.

1. Internet Explorer MSXML2 4.0 parser – No errors or warnings were reported in either mode.

2. Brown University Scholarly Technology Group's XML Validation Form [6] – Reported "Document validates OK" in either mode.

3. Richard Tobin's XML well-formedness checker and validator [7] – Reported that the document appears to be well-formed and listed no validity or namespace errors in either mode.

## Conclusion

A user extensible DTD has been written for the API TMA DES and is in routine use in our ACSR program. Improvements to the TMA DES have been suggested by our experience in importing other institution's exported TMA data. Enhancements to the TMA DES are suggested. Better specified identifiers and hierarchical relationships among blocks, slides and cores will facilitate automatic use of the data. Proposed improvements will remove the potential for some ambiguous situations and strengthen the ability to understand data using this format. Our tool can be used to reorder data and add missing identifiers; upgrading data from the original specification to the improved can be automatically accomplished. Using a DTD (optionally reflecting our proposed enhancements) can provide stronger validation of exported TMA data. We hope interested parties will continue to participate in the evolution of the API TMA DES.

## Availability and requirements

Example TMA DTD and XML data files and all source code for the *BrowseTMA *tool and related software are available at the public ACSR Mid-Region web site . Personal computers running Microsoft Windows 98, 2000, NT and XP have been used with Internet Explorer 6.0, Word 2002, and FrontPage 2002 in this work.

## List of abbreviations used

ACSR – AIDS and Cancer Specimen Resource

AIDS – Acquired Immunodeficiency Syndrome

AMB – AIDS Malignancy Bank

API – Association for Pathology Informatics

CDE – Common Data Elements

DCTD – Division of Cancer Treatment and Diagnosis

DES – Data Exchange Specification

DTD – Document Type Definition

HIV – Human Immunodeficiency Virus

HTML – Hypertext Markup Language

LDE – locally defined data elements

NCI – National Cancer Institute

OSU – Ohio State University

TMA – Tissue Microarray

TMA DES – Tissue Microarray Data Exchange Specification

URI – Universal Resource Identifier

W3C – World Wide Web Consortium

XML – Extensible Mark-up Language

XSL – Extensible Stylesheet Language

XSLT – XSL Transformation

## Competing interests

The author(s) declare that they have no competing interests.

## Authors' contributions

DGN developed TMA DES DTD and wrote the first draft of the manuscript. LWA is the principle investigator for this project and participated in writing the manuscript. Both authors reviewed and commented on successive drafts of the manuscript and versions of the software and have approved the final manuscript.

## Pre-publication history

The pre-publication history for this paper can be accessed here:



## Supplementary Material

Additional File 1 All additional files are available at . 
This .zip file contains the following eight files which may be extracted using standard unzip software. 
**Sample specification TMA export: TA00-050.xml**: This TMA block contains 35 cores in a 5 by 7 array. This XML file can be viewed with Internet Explorer or another browser or editor. 
**Sample improved TMA export: TA00-050_reordered.xml**: This XML file is a modified version of TA00-050.xml. Certain identifier CDEs (filename, block_identifier, slide_identifier, core_array-id) have been moved to the first position inside the parent CDE. Identifiers have been added where not present. This file can be used with the DTD in improved mode. 
**TMA DES export DTD: tmades.dtd**: This DTD defines the TMA DES CDEs (tags) and allowed structure of API TMA DES exports. Also present are elements to define the allowed structure of TMA styles in TMA style files. It can be viewed with any text editor. 
**TMA local data element file DTD: myLDEs.dtd**: This file defines the LDEs (tags) and their allowed structure. It can be viewed with any text editor. 
***BrowseTMA* Reorder XSLT script: BrowseTMAReorder.xsl**: This XSLT file reads in a TMA block XML file and produces another version . In the resulting XML file, certain identifier CDEs (filename, block_identifier, slide_identifier, core_array-id) have been moved to the first position inside the parent CDE. Identifiers have been added where not present. The resulting file will use the DTD in the improved mode. 
***BrowseTMA* WSH batch file: BrowseTMAReorder.bat**: This WSH batch file runs the BrowseTMAReorder.xsl XSLT script and then the fixOrdered.js for a TMA block. The resulting file will use the DTD in the improved mode. 
**Fix ordered XML JScript: fixOrdered.js**: This JScript program reads a passed XML file and makes a repaired version. 
**XSLT test JScript: xsltTest.js**: This JScript program runs the Microsoft XSLT Parser, MSXML2 4.0, on a passed XML file and XSLT file.  Click here for file
